# Preclinical Pharmacokinetics and Translational Pharmacokinetic/Pharmacodynamic Modeling of M8891, a Potent and Reversible Inhibitor of Methionine Aminopeptidase 2

**DOI:** 10.1007/s11095-023-03611-z

**Published:** 2023-10-05

**Authors:** Floriane Lignet, Manja Friese-Hamim, Frank Jaehrling, Samer El Bawab, Felix Rohdich

**Affiliations:** 1 Drug Discovery Technologies, the Healthcare Business of Merck KGaA, 250 Frankfurter Strasse, 64293 Darmstadt, Germany; 2 Research Unit Oncology, the Healthcare Business of Merck KGaA, Darmstadt, Germany; 3 Translational Medicine, the Healthcare Business of Merck KGaA, Darmstadt, Germany; 4 Present Address: Translational Medicine, Servier, Suresnes, France

**Keywords:** allometry, human PK prediction, PK/PD, translational modeling

## Abstract

**Introduction:**

M8891 is a selective and reversible inhibitor of methionine aminopeptidase 2 (MetAP2). We describe translational research to define the target pharmacokinetics (PK) of M8891 and associated pharmacodynamic (PD) levels, which were used to support efficacious dose selection in humans.

**Methods:**

*In vitro* and *in vivo* PK characteristics were investigated in animal species, and data integrated using *in vitro–in vivo* correlation and allometric methods to predict the clearance, volume of distribution, and absorption parameters of M8891 in humans. In parallel, inhibition of MetAP2 activity by M8891 was studied in renal cancer xenografts in mice by measuring accumulation of Met-EF1α, a substrate of MetAP2. The corresponding PD effect was described by a turnover and effect compartment model. This model was used to simulate PD at the M8891 dose showing *in vivo* efficacy, i.e. significant tumor growth inhibition. Simulations of M8891 PK and associated PD in humans were conducted by integrating predicted human PK parameters into the preclinical PK/PD model.

**Results:**

The target minimum PD level associated with efficacy was determined to be 125 µg Met-EF1α per mg protein. Integrating predicted human PK parameters into the preclinical PK/PD model defined a minimal M8891 concentration at steady-state (C_trough_) of 1500 ng/mL (3.9 µM) in humans as being required to produce the corresponding minimum target Met-EF1a level (125 µg per mg protein).

**Conclusion:**

The defined target PK and PD levels supported the design of the clinical Phase Ia dose escalation study of M8891 (NCT03138538) and selection of the recommended Phase II dose.

**Supplementary Information:**

The online version contains supplementary material available at 10.1007/s11095-023-03611-z.

## Introduction

Methionine aminopeptidase 2 (MetAP2) is one of two known cytoplasmic isoforms that proteolytically cleave the N-terminal methionine from nascent proteins, thereby facilitating their intracellular translocation, stability, and protein–protein interactions. Inhibition of the enzymatic activity of MetAP2 leads to a variety of changes in protein functionality impacting both tumor growth and proliferation, as well as, by acting directly on endothelial cells, inhibiting angiogenesis in a vascular endothelial growth factor (VEGF)-independent manner. Thus, MetAP2 inhibition has potential as a minimally toxic, non-competing therapy that could complement current targeted therapies such as those inhibiting the VEGFR pathway or cytotoxic drugs [1–4].

M8891 is an orally bioavailable molecule designed to inhibit MetAP2 selectively and reversibly [5]. In contrast to covalently binding (irreversible) MetAP2 inhibitors belonging to the natural product fumagillin derivatives, initially identified as potent antiangiogenic molecules [6–9], M8891 represents a novel class of MetAP2 inhibitor. It does not show any structural similarity with fumagillin, nor does it covalently bind the target protein or require parenteral application.

While M8891 showed moderate activity as monotherapy in a variety of different preclinical tumor models, increasing and long-lasting responses were observed in combination with agents such as multi-targeted or VEGFR-specific tyrosine kinase inhibitors (TKIs) [1, 10]. While slowing tumor growth initially, long-term tumor stasis can be observed in later phases of treatment. M8891 is thus hypothesized to synergize with selected therapies through deepening and extending the duration of response by impacting cellular processes in tumor and tumor-associated endothelial cells. In addition, *in vitro* knock-out data and *in vivo* xenograft model results strongly suggest that MetAP2 inhibition has a more profound impact in a p53 functional wild-type background [10].

The selection of the correct doses in first-in-human studies, but also the early identification of a target plasma concentration and associated pharmacodynamic (PD) levels associated with efficacy, are critical for the rapid and efficient development of therapeutics in humans. Translational modeling and simulation of preclinical data have become key tools to determine the link between pharmacokinetics (PK), PD modulation, and efficacy, and to infer a potential efficacious dose range [11–13].

Herein, we describe a translational PK/PD/efficacy modeling approach used to define target PK and PD levels to be used to inform the design of a clinical Phase Ia dose-escalation study of M8891 (NCT03138538) and support the selection of the recommended Phase II dose (RP2D).

## Materials and Methods

### *In Vitro* ADME

#### Plasma Protein Binding

*In vitro* protein binding of M8891 was investigated at concentrations of 0.5, 5, and 15 µM by equilibrium dialysis (3 h) in human, dog, monkey, rat, and mouse plasma, and in a solution of 600 µM purified human serum albumin (HSA) and 20 µM purified α1-acid glycoprotein (AAG) in 70 mM phosphate buffer, pH 7.4, respectively. Concentrations of M8891 were determined by liquid chromatography coupled with tandem mass spectrometric detection (LC–MS/MS; for details see section Bioanalytics). Warfarin was used as a control.

#### Blood-To-Plasma Ratio

The *in vitro* distribution of M8891 was investigated in fresh human, dog, monkey, rat, and mouse whole blood. To maintain physiological conditions, the pH of fresh whole blood was stabilized by the addition of 5% (v/v) (for monkey, rat, and mouse) or 7% (v/v) (for human and dog) phosphate buffer pH 7.4 (70 mM), which was considered during evaluation. M8891 was spiked into pH-stabilized whole blood of human, monkey, dog, rat, and mouse at concentrations of 0.2 and 2 μM, respectively. Subsequently, plasma was separated from blood cells by centrifugation and aliquots were taken from plasma for quantification of test item concentration by ultra-high performance LC–MS/MS (UPLC-MS/MS) and for calculation of the blood-to-plasma ratio. The distribution of the internal standard MSC1326749A-1 at 0.2 and 2 μM was investigated as a positive control to demonstrate the validity of the applied test system and experimental procedure.

#### Caco-2 Permeability

The apparent permeability (P_app_) of M8891 was determined in Caco-2 cells (TC-7 clone, supplied by INSERM U-505, Paris, France; RRID: CVCL_0233) at a concentration of 1 µM. Cell monolayers were allowed to differentiate for 14 days in culture before the experiment. On the day of the experiment, cell confluency was evaluated by measurement of trans-epithelial electrical resistance. The compound was dosed on the donor side and analyzed by LC–MS/MS in the donor as well as receiver wells after 2 h of incubation. Permeability was determined in the apical-to-basolateral (A > B) and in the basolateral-to-apical (B > A) direction using a pH of 7.4 in both compartments.

#### Metabolic Stability

##### In liver Microsomes

The *in vitro* metabolic stability of M8891 was investigated in liver microsomes at a substrate concentration of 1 µM. The decline of the parent drug up to 30 min was quantified by LC–MS/MS, and the *in vitro* CL_int_ was expressed as μL/min/mg protein and corresponds to the volume of the incubation medium that is cleared from drug. Unbound intrinsic clearance (CL_u,int_) values were derived by correcting for liver microsome binding (estimated using the equation published by Hallifax and Houston [14]).

##### In Hepatocytes

The *in vitro* metabolic stability of M8891 was also investigated in cryopreserved hepatocytes at a substrate concentration of 5 µM in the presence of 5% plasma of each species. The decline of the parent drug up to 5 h was quantified by LC–MS/MS. The *in vitro* CL_int_ was expressed as μL/min/10^6^ cells and corresponds to the volume of the incubation medium that is cleared from drug. CL_u,int_ values were derived by correcting for measured hepatocyte binding and plasma protein binding scaled to 5% plasma.

### *In Vivo* Pharmacokinetics

#### *In Vivo* PK In-Life Phase

Single-dose PK studies in mouse, rat, dog, and monkey were performed at Nuvisan GmbH (Grafing, Germany) according to established practices and operating procedures.

These animal experiments were approved by the District Government of Upper Bavaria and conducted in compliance with German and European Animal Welfare Laws and Regulations in an Association for Assessment and Accreditation of Laboratory Animal Care (AAALAC)-accredited facility.

Single-dose intravenous (i.v., 0.2 mg/kg) and oral (p.o., 0.5 mg/kg) PK studies with M8891 were performed in NMRI mice (n = 3 per dose group), Beagle dogs (n = 2 per dose group), and cynomolgus monkeys (n = 3). Wistar rats (n = 3 per dose group) were dosed with M8891 at 2 mg/kg i.v. and 5 mg/kg p.o., respectively. A solution of PEG200 40% in water was used for both i.v. and the p.o. routes. For these standard PK assessments, the size of each dose group was selected to allow variability of exposure to be captured while minimizing animal use.

Pre-dosing blood samples (dog and monkey) as well as multiple consecutive samples up to 24 h post-dose (all species) were taken and used for plasma isolation.

#### Bioanalytics

Details of the bioanalytic methods used can be found in the Supplementary Materials.

#### PK Evaluation (Non-Compartmental Analysis)

Maximum plasma concentration (C_max_) and time to reach maximum plasma concentration (t_max_) were taken from the observed data. Area under the curve (AUC), clearance (CL), volume of distribution at steady-state (V_ss_), and elimination half-life (t_1/2_) were calculated by non-compartmental analysis (NCA) using the custom-made software “DDS-Tox” developed by Nuvisan GmbH, which provides results comparable to Phoenix WinNonlin (Certara, L.P., Princeton, New Jersey, USA). AUC values were calculated by NCA using the linear up/log down trapezoidal method. More details on calculation methods can be found in the Supplementary Materials.

### Estimation of The Human PK Parameters of M8891

#### *In Vitro* Scaling

Human and animal *in vitro* CL_u,int_ values as determined above (see metabolic stability) were scaled to the *in vivo* CL_int_ using physiological scaling factors, i.e. the *in vitro* CL_u,int_ was multiplied by the number of hepatocytes/g liver or mg protein liver microsomes and g liver/kg of body weight (extracted from Simcyp™ V12.1, Certara, and [15]). The *in vivo* CL was predicted from the scaled CL_int_ by applying a parallel-tube blood-flow model that incorporates plasma protein binding and blood/plasma ratios (B/P) [16, 17], as described in the equation below:

1 $${CL}_{H}=\frac{{Q}_{h}\times fu \times {CL}_{int}}{{Q}_{h}+fu \times {CL}_{int}/(B/P)}$$

#### Allometric Scaling

The CL and V_ss_ of M8891 in human were estimated based on preclinical *in vitro* and *in vivo* PK data obtained in 4 species using allometric scaling approaches corrected for protein binding and CL_int_ (CL-NAS_fub_, V-SA_fub_) [18–21]. The input parameters CL and V_ss_ for the preclinical species were derived from the individual i.v. PK profiles by NCA. The prediction of human clearance also considered inter-individual differences in metabolic stability, i.e. CL_int_ in liver microsomes to normalize the input parameters.

Additionally, the CL was estimated using the Tang-Mayersohn equation, which is based on cross-species allometry and the ratio between human and rat protein binding [22], and the rule of exponent method (RoE), which selects the most appropriate method based on the value of the exponent derived by simple allometry (SA) [23].

Predictions of the volume of distribution were also made using the Øie-Tozer model, using physiological constants derived from Obach *et al.* [24] and the human–dog proportionality model as defined by Eq. (2).

2 $${V}_{hu}={V}_{dog}\times {~}^{{f}_{u,hu}}\!\left/ \!{~}_{{f}_{u,dog}}\right.$$

#### Physiologically Based Pharmacokinetic (PBPK) Modeling

GastroPlus® (version 9.5, SimulationsPlus, Lancaster, CA, USA) was used to establish a PBPK model of M8891 using PK data from rat and dog and the compound-specific parameters, i.e. physico-chemical properties, *in vitro* CL_int_, permeability, fraction unbound, and B/P ratio data. Using an iterative approach, the model was built and verified against the observed preclinical exposure to define first the distribution and elimination characteristics, then the oral absorption processes. The validated model was used to estimate human CL and V_ss_ as well as to predict the rate and extent of absorption of M8891. A full description of the model development can be found in the Supplementary Materials.

### Translational Preclinical *In Vivo* Studies

All procedures in animals described below were performed in compliance with the Animal Welfare Act(s), the recommendations of AAALAC, and national animal health regulations. Animal protocols were reviewed by the Animal Welfare Body of the relevant test facility of Merck Healthcare KGaA (Darmstadt, Germany) and approved by the local authority.

For all *in vivo* studies, human Caki-1 renal tumor cells (ATCC: HTB-46, RRID: CVCL_0234; 5 million in 100 µL PBS w/o Ca/Mg, Matrigel [1:1]) were subcutaneously inoculated into the right flank of female 5–6-week-old CD-1 nude mice provided by Charles River Laboratories (CRL), Sulzfeld, Germany. Tumor growth was observed over time by measuring tumor length (L) and width (W) twice weekly using calipers. Tumor volume was calculated using the formula LxW^2^/2. Data were collected in Study Advantage™ (data collection system).

PD biomarker analysis was performed at Indivumed GmbH, Hamburg, Germany. The Simple Western™ Size (SallySue, ProteinSimple, USA) method for the detection of total elongation factor 1α (EF1α-1) (Abcam, # ab186386, 0.2 mg/ml) and of methionine EF1α (Met-EF1α) (antibody clone MKV-3–165-11, Lot 025Y14M3, 0.2 mg/mL) was used to analyze preclinical xenograft tumor tissues. EF1α has been identified as a substrate of MetAP2 in proteomics studies, and pharmacological inhibition of MetAP2 leads to dose-dependent accumulation of uncleaved Met-EF1α in tumors, thereby qualifying it as a valid pharmacodynamic (PD) target engagement biomarker [1, 10]. Tissues were homogenized using Precellys-24 (Bertin Technologies, France) and total protein concentration was determined using the BCA Protein Assay Kit (Thermo Scientific, # 23225). Tumor lysates were separated in capillaries by size, immobilized to the capillary wall via UV, incubated with primary and horseradish peroxidase (HRP) -conjugated secondary antibodies, and detected by chemiluminescence. The molecular size for immunodetected proteins as well as signal intensity, area under the curve were analyzed and reported using the Compass software. Total and Met-EF1α-1 were quantified along a standard curve for recombinant total EF1α-1 protein (Abcam, # ab177675, 1 mg/ml) measured in each experiment and normalized to total protein concentrations.

#### PK/PD Studies

##### Study PK/PD-01 and PK/PD-02

When Caki-1 tumors reached a size of 300–500 mm^3^, mice were randomized into treatment groups (n = 5) that received either vehicle control or M8891 in a solution of 0.25% Methocel in MilliQ water at various doses. CD1 nu/nu mice were treated with a single administration (PK/PD-01), or four daily administrations (PK/PD-02) of M8891 p.o. at 10, 25, or 100 mg/kg, respectively. At designated time points (1 h, 7 h, 24 h, 48 h, 72 h, and 96 h) following treatment, animals were euthanized, and plasma and tumor tissues were collected, snap frozen, and processed for PK and PD analysis as described above.

#### Efficacy Studies

##### Study EFF-01

Anti-tumor efficacy and PD study. When Caki-1 tumors reached a size of 95–250 mm^3^, CD1 nu/nu mice were randomized into treatment groups (n = 10) that received either vehicle control or M8891 at different doses, using a solution of 0.25% Methocel in MilliQ water. Mice were treated for 6 weeks with daily administrations of M8891 p.o. at 10, 25, or 100 mg/kg. At designated time points (1 h, 7 h, and 24 h) following treatment, animals were euthanized and plasma and tumor tissues were collected, snap frozen, and processed for PK and PD analysis as described above.

##### Study EFF-02

Anti-tumor efficacy study. When Caki-1 tumors reached a size of 100–150 mm^3^, CD1 nu/nu mice were randomized into treatment groups (n = 10) that received either vehicle control or M8891 at different doses, using a solution of 0.25% Methocel in MilliQ water. Mice were treated for 6–7 weeks with bi-daily administrations of M8891 p.o. at 10, 25, or 50 mg/kg. PD analysis was not performed in this study.

### Modeling

#### PK/PD Modeling of Caki-1 Xenograft Data

The PK/PD analyses and simulations were performed using Phoenix WinNonlin 6.4 (Certara, L.P., Princeton, New Jersey, USA). Model selection was based on of the following criteria: objective function value (-2LL); Akaike Information Criteria (AIC); Bayesian Information Criteria (BIC); the precision of the parameter estimates (CV%); and diagnostic plots.

The PK/PD modeling and estimation of PD level associated with efficacy in the Caki-1 xenograft model were performed in three steps. Step 1: the data from the single and repeated dose PK/PD studies in mouse (PK/PD-01 and PK/PD-02) were pooled to fit a mouse PK model. Step 2: the Met-EF1α data from the single and repeated dose PK/PD studies (PK/PD-01 and PK/PD-02) were pooled to fit a PD model, using plasma concentrations simulated with the PK model developed at the previous step. Step 3: the model was validated by simulating the Met-EF1α modulation at the doses used in efficacy study EFF-01 and compared to the measured values. Thus, the model could be used to identify the extent of Met-EF1 modulation by M8891 administered at efficacious doses.

#### PK Model Structure

An oral one-compartment model with first order absorption and elimination was fitted to the PK data from single- and repeated-dose Caki-1 studies PK/PD-01 and PK/PD-02. The PK model could be described with the following equations:

3 $$C\left(t\right)=\frac{{k}_{a}\times Dose}{{(k}_{a}- {k}_{e}) \times V/F } \times \left({e}^{-{t.k}_{e}}-{e}^{-{t.k}_{a}}\right)$$

4 $${k}_{e}=\frac{CL}{V}$$

where C(t) represents plasma concentration at time t after drug administration, k_a_ absorption rate constant, F oral bioavailability, k_e_ elimination rate constant, V volume of distribution, and CL total body clearance.

#### PD Model Structure

The effect of M8891 on Met-EF1α level was described by an effect compartment and turnover model according to Eqs. (5) and (6).

5 $$\frac{{dC}_{e}}{dt} = {k}_{e0}\times (C-{C}_{e})$$

6 $$\frac{dE}{dt} = {k}_{in}- {k}_{out}\times E \times \left(1-\frac{{I}_{max}\times {C}_{e} }{{C}_{e}+{IC}_{50}}\right)$$

**Fig. 1:**
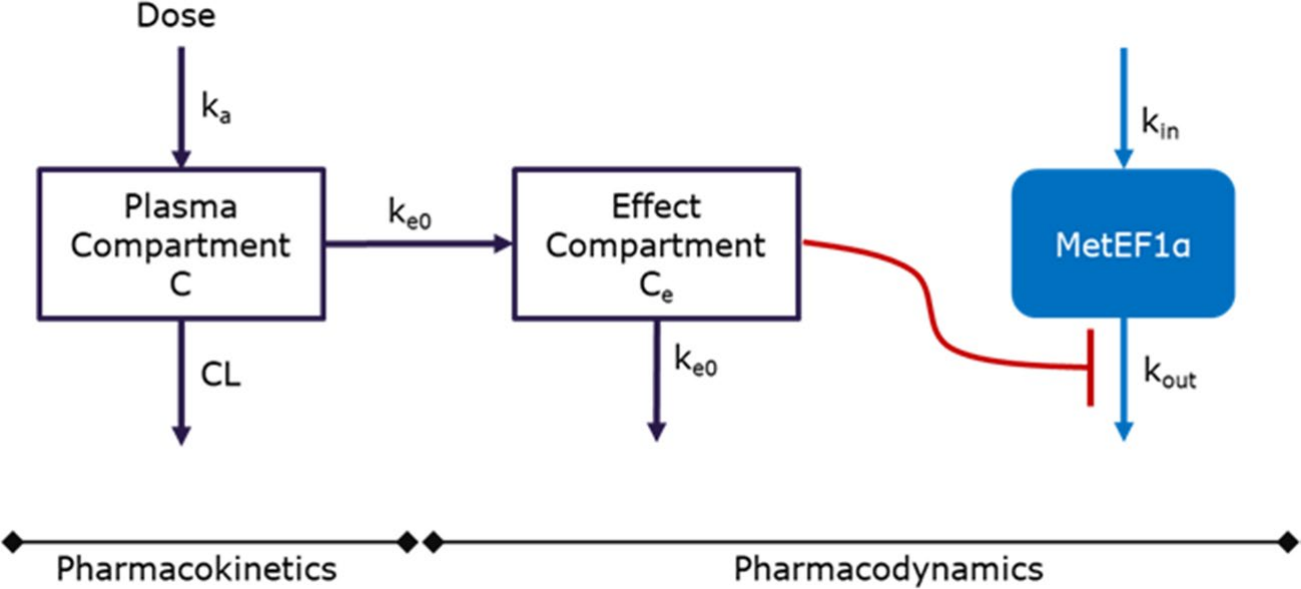
Structure of the PK/PD model. The PK of M8891 is described by a one-compartment model with a first-order absorption rate k_a_ and a clearance CL. The PD model includes an effect compartment characterized by a first-order in- and outward distribution rate k_e0_, and a turnover model for Met-EF1α, with a zero-order synthesis rate k_in_ and a first-order degradation rate k_out_

In these equations, C_e_ represents drug concentration in the effect compartment, k_e0_ first-order rate constant for transport into the effect compartment, I_max_ maximal inhibitory effect, IC_50_ concentration in effect compartment producing half of the maximum effect, and E effect, here the level of Met-EF1α. A schematic representation of the PK/PD model is given in Fig. 1.

#### Human PK and PD Prediction

The human oral PK profile was simulated using a one-compartment PK model based on the predicted human i.v. PK parameters for the terminal arm, whereas the ascending oral arm was established via PBPK modeling. The previously established xenograft PK/PD model, with the PK part replaced by the predicted human PK model, was used to simulate Met-EF1α modulation after M8891 dosing in humans. The human doses yielding a target Met-EF1 level established from the Caki-1 xenograft studies and the associated minimal M8891 concentration (C_trough_) in human plasma were estimated by simulations.

## Results

### Absorption, Distribution, Metabolism, and Excretion (ADME) and *In Vivo* PK Profiles

#### *In Vitro* characteristics

##### Protein Binding and Whole Blood Distribution

M8891 exhibited relatively high plasma protein binding in all species, with similar values at 0.5 and 5 μM, and a mean fraction unbound (fu) at the expected therapeutic concentration (5 μM) of ≈2–5% in mouse, dog, monkey, and humans, and a slightly higher fu in rat of ≈10%. The data further suggested that both HSA and AAG contributed to the overall binding of M8891 in human plasma, with a mean fraction unbound at 5 μM of 4.5% and 4.9% in the HSA and the AAG solutions respectively. The mean human fu in plasma was determined to range from 1.3 to 2.8%, with a median of 1.8% (Table I). The measured fraction unbound of control warfarin (fu = 1.7%) was in the expected range, confirming the validity of the experimental system.

**Table I:** CL_int_, Protein Binding (Fraction Unbound) and Blood-to-Plasma Ratios of M8891 used for Allometric Scaling and GastroPlus Simulations

	Unbound CL_int_		Protein binding^a^	Whole blood distribution^b^
	Hepatocytes	Liver microsomes^c^	Plasma	Blood
Species	µL/min/10^6^ cells	µL/min/mg prot	median fu (%)	B/P ratio
Mouse	120	60	3.2	0.71
Rat	30	29	9.6	0.74
Monkey	3.8	19	4.4	0.73
Dog	3.6	11	1.9	0.59
Human	6.0	12	1.8	0.58

1): Data were obtained at 0.5, 5, and 15 µM, and are reported as the median of the 3 data sets

2): Data were obtained at 0.2 and 2 µM, and are reported as the mean of the 2 data sets

3): Data were obtained in 0.5 mg/mL microsomal protein (P) and extrapolated to unbound values using the equation F_u,inc_ = 1/(1 + P × 10 ^0.072∙logD2+0.067∙logD – 1.126^), as described by Hallifax and Houston in [14]

Furthermore, at the tested concentration range (0.2 and 2.0 μM), low binding to blood cells and preferential distribution into the plasma were observed with no concentration-dependency, in agreement with the *in vivo* results described above, and driven by high binding to plasma proteins. The calculated blood-to-plasma concentration ratios, based on measured hematocrit values and plasma concentrations, were comparable in all tested species (Table I) and ranged between 0.74 (rat) and 0.58 (human). In each species, the distribution of the positive control MSC1326749A-1 in the plasma fraction was in a reasonable range compared to historical data, validating the test system.

##### Permeability and Solubility

The compound showed no significant pH-dependent solubility, with moderate solubility under acidic conditions (≈27 μg/mL in SGF media; pH 1.2) and slightly higher solubility at pH values of ≥ 6 and in intestinal fluids (i.e. ≈54 μg/mL in FaSSIF media) (Table S1). The apparent permeability (P_app A,B_) of M8891 in Caco-2 cells in the absence of cyclosporine A (P-glycoprotein (P-gp) inhibitor) was found to be high (35.7 × 10–6 cm/s) and no significant active efflux (ratio: 1.2) was observed. The passive permeability (P_app A,B_) in the presence of cyclosporine A was 39.9 × 10–6 cm/s (Table S2).

##### Metabolic Stability

**Fig. 2:**
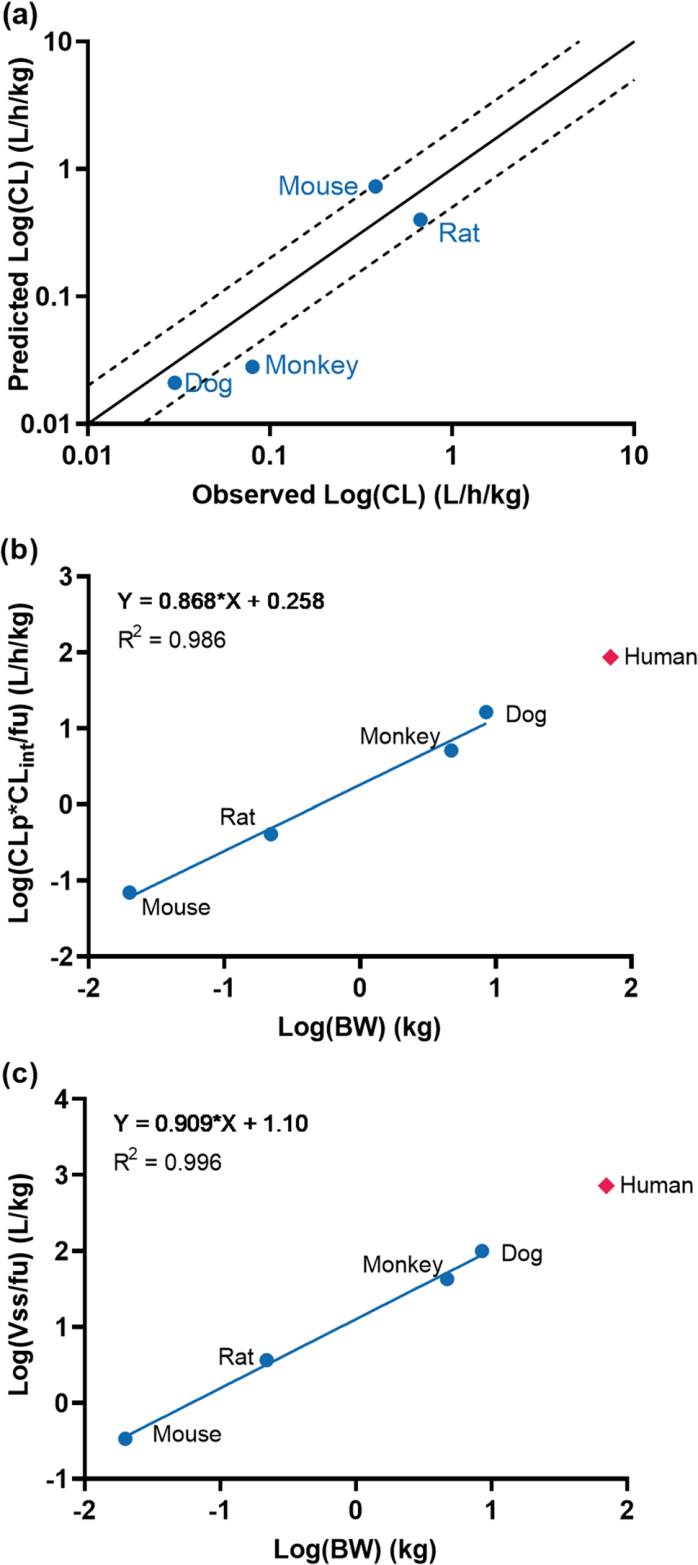
**(a)** *In vitro* to *in vivo* correlation of M8891 total clearance based on scaling of CL_int_ in hepatocytes of mouse, rat, dog, and monkey. Solid line: unity line; dashed lines: twofold interval. **(b)** Prediction of CL (human) of M8891 based on NAS_fub_ (4 species allometry including *in vitro* correction factors) [18]. **(c)** Prediction of V_ss_ (human) of M8891 based on SA_fub_ (4 species allometry including *in vivo* correction factors) [21]

The metabolic stability of M8891 was assessed in liver microsomes and in hepatocytes from mice, rats, dogs, monkeys, and humans complemented with 5% plasma of each species as matrix. The drug was shown to be metabolized with low-to-moderate CL_u,int_ ranging from 12 to 60 μL/min/min and 6.0 to 120 μL/min/10^6^ cells, respectively (Table I). The hepatocyte values were scaled to the total body clearance for each species considering plasma protein binding, hepatocyte binding, and blood-to-plasma ratio. These scaled clearances corresponded to predicted hepatic *in vivo* CLs ranging from 0.01 L/h/kg (human) to 0.73 L/h/kg (mouse), or approximately 1 to 21% of the respective liver blood flows and were thus in the low-to-medium CL range. Accordingly, hepatocytes of four animal species predicted the *in vivo* clearance of M8891 (see below) within 0.52- and 2.9-fold (Fig. 2a). Based on this, it can be assumed that the drug is predominantly eliminated by metabolic hepatic clearance, and that clearance predictions for humans can be performed based on CL_int_ data.

### *In Vivo* PK

Intravenous M8891 exhibited favorable *in vivo* PK properties in mice, rats, dogs, and cynomolgus monkeys. In all species investigated, M8891 showed low CL (ranging from 0.03 to 0.4 L/h/kg, corresponding to 1 to 6% of the liver blood flow), small to medium V_ss_ (ranging from 0.23 to 1.3 L/kg), and medium-to-high oral bioavailability (F, ranging from 40 to 80%). Detailed data can be found in Table II.

**Table II:** Body Weights and Pharmacokinetic Parameters of M8891 in Preclinical Species Obtained by Non-Compartmental Analysis

	Body weight	Plasma clearance	Volume of distribution at steady-state	Bioavailability
**Species**	**(kg)**	**(L/h/kg)**	**(L/kg)**	**(%)**
Mouse	0.029	0.378	0.37	38
Rat	0.270	0.350	1.30	72
Monkey	4.60	0.078	0.41	68
Dog	8.36	0.034	0.23	80

### Human PK Parameters Prediction

Prediction of the human plasma CL and V_ss_ was based on preclinical *in vivo* data for female mice, rats, beagle dogs, and cynomolgus monkeys by using allometric scaling approaches corrected for protein binding and considering interspecies differences in CL_int_ in liver microsomes. In addition, a PBPK modeling approach was used to determine CL and V_ss_, which is described in more detail in the section below. The results of the various methods utilized are presented in Table III.

**Table III:** Predicted Human Pharmacokinetic Parameters of M8891 Using Different Approaches

Clearance		Volume of distribution		T_1/2_
(L/h/kg)	Method	(L/kg)	Method	(h)
0.022	NAS_fub_	0.186	SA_fub_	5.9
0.018	TME	0.216	OT	8.3
0.020	SA/RoE	0.215	Human–dog proportionality	7.4
**0.02 ± 0.002**	**Mean ± SD**	**0.21 ± 0.017**	**Mean ± SD**	**7.3**
0.014	PBPK	0.240	PBPK	14
0.01	IVIVE	0.240	PBPK	17

NAS_fub_, Allometric scaling normalized by median fu and corrected by CL_int_ [18]; SA, Simple allometry; RoE, Rule of exponents [23]; TME, Tang-Mayersohn-Eq. (22); OT, Øie-Tozer [24]; SA_fub_, simple allometry normalized by fu [20]; PBPK, physiologically based pharmacokinetics; IVIVE, *in vitro-in vivo* extrapolation from hepatocyte data

Bold entries indicate that the mean of the first three methods were used for the final human simulations

The allometric scaling approaches for clearance (CL-NAS_fub_) and volume of distribution (V-SAfub) are illustrated in Fig. 2b and c, respectively, using the PK parameters of the preclinical species as input.

Using 4-species allometric scaling with both CL_int_ in microsomes as well as plasma protein binding (at 5 μM) as correction factors (CL-NAS_fub_), a low plasma CL (CL_P_) of 0.022 L/h/kg in humans was predicted. The correlation coefficient of the linear regression (R^2^) was 0.986 (Fig. 2b).

Four-species allometry in turn corrected for plasma protein binding predicted V_ss_ of M8891 of 0.186 L/kg, which is assumed to be in the low range, i.e. lower than the total body water in humans. As shown in Fig. 2c, the correlation coefficient of the linear regression (R^2^) was 0.979.

Based on these results, means of the human CL and V_ss_ from individual scaling methods were considered as meaningful input parameters for PK modeling and simulations and were estimated to be 0.020 ± 0.002 L/h/kg and 0.21 ± 0.017 L/kg, respectively. Based on the CL value, first-pass extraction in the liver was estimated to be ≈2% (human liver blood flow Q_h_ = 1.16 L/h/kg), whereas first-pass metabolism/elimination in the gut was considered to be negligible. Moreover, the CL value predicted by the allometric scaling methods described above was in line with the CL value extrapolated from the *in vitro* CL_int_ value, i.e. 0.01 L/h/kg (twofold) generated in human hepatocytes, thus confirming both the hepatic elimination route and the CL prediction by allometric scaling of M8891.

Using the predicted PK parameters CL and V_ss_ as input, a plasma i.v. t_1/2_ of ≈7.3 h [6–17] was calculated according to the equation t_1__/2_ = ln(2) x CL / V_ss_.

#### PBPK Modeling

An extensive PBPK including compartmental modeling approach has been performed as described in detail in the Supplementary Materials. For this approach, a complete compound-specific *in vitro* data set was generated, i.e. physico-chemical and formulation properties (Table S1), permeability data (Table S2), CL_int_ data (Table I), fractions unbound, and B/P ratios (Table I). This was used as the input, together with the system parameters, implemented in GastroPlus® (CSP, Table S8). Based on this, the i.v. CL in rats and dogs as well as V_ss_ in dogs could be predicted well via PBPK modeling, whereas the rat Vss was predicted to be almost fourfold lower compared to the observed value (Table S3). Accordingly, the simulation output by PBPK modeling shows some deviations in rats at later time points (Figure S1). In addition, further simulations via compartmental PK modeling were performed with the rat and dog i.v. PK data, and a one-compartment model was found to describe the drug’s disposition with reasonable accuracy. This model was further applied to simulate the oral concentration *vs.* time (C/t) profile courses of M8891 in rats and dogs in order to evaluate the accuracy of estimation of the oral absorption process and first-pass effect. Respective simulations indicated that predictions based on a compartmental approach described the observed oral C/t with reasonable accuracy (Figure S4).

Following this validation step, the PBPK model was used to simulate the human PK of M8891. The predicted values for human i.v. CL and V_ss_, i.e. 0.014 L/h/kg and 0.24 L/kg, estimated by PBPK modeling only, were close to the values estimated via the scaling methods described above (Table III). In addition, oral bioavailability (F) was predicted to be at least 60% and the rate of absorption (k_a_) was estimated to be 0.35h^−1^. More details of the PBPK modeling approach can be found in the Supplementary Materials.

### Preclinical PK/PD/Efficacy Modeling

#### PK/PD Modeling

**Fig. 3:**
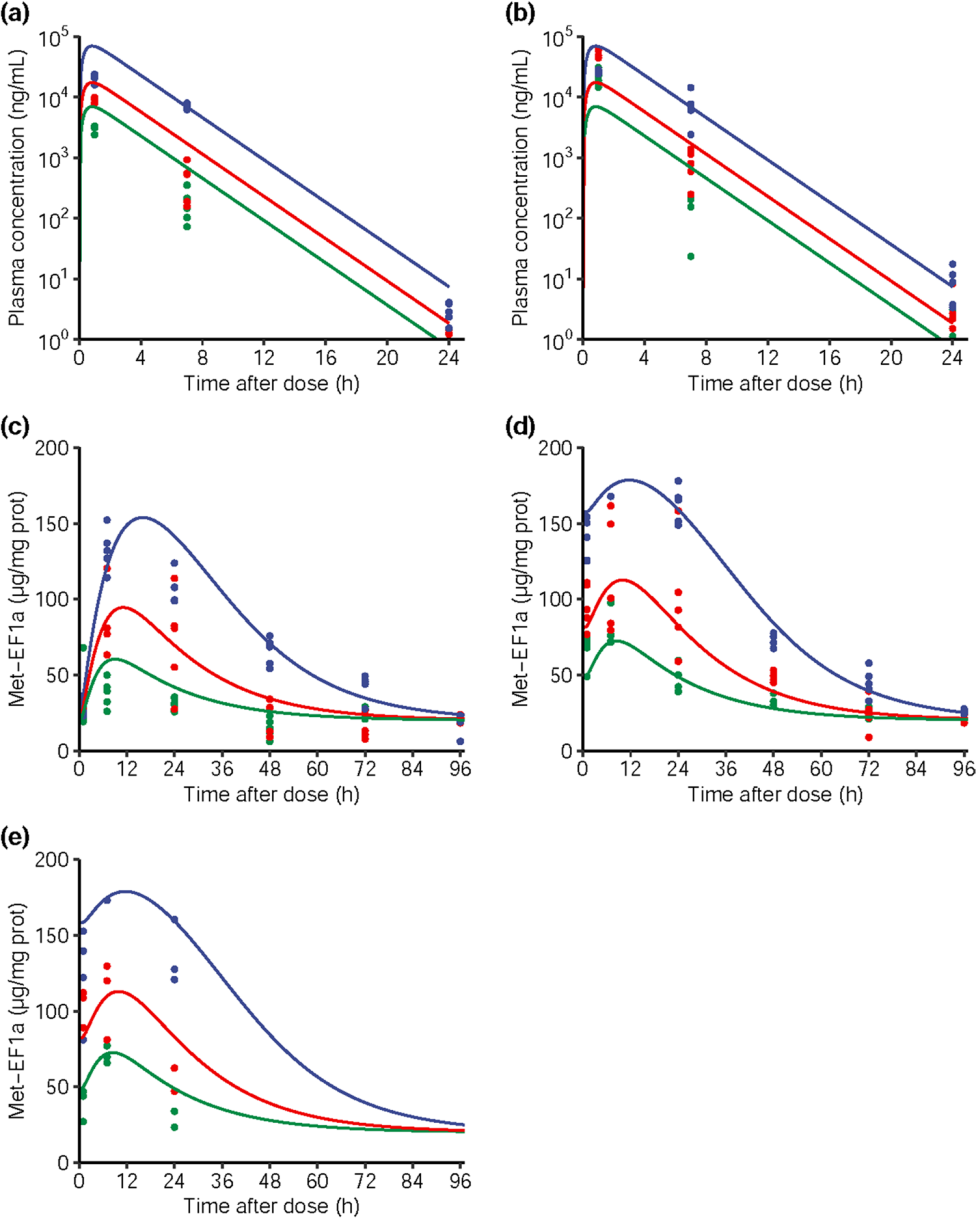
**(a-b)** Overlay of M8891 plasma concentrations measured in mice after oral administration (points) and predicted by a fitted model (solid lines) at 10 mg/kg (green), 25 mg/kg (red), or 100 mg/kg (blue) **(a)** after a single dose and **(b)** after the last of four daily doses. **c-e**: Overlay of Met-EF1α levels measured in Caki-1 xenografts after oral administration of M8891 (points) and predicted Met-EF1α levels predicted by the model (solid lines) at 10 mg/kg (green), 25 mg/kg (red), or 100 mg/kg (blue) **(c)** after a single dose, study PK/PD-01; **(d)** after the last dose of 4 daily doses, study PK/PD-02; and **(e)** after the last dose (day 42) of repeated daily oral administration of M8891, study EFF-01

Exposure data after single and repeated administration of 10, 25, and 100 mg/kg of M8891 could be well described by a one-compartment model with first order absorption and elimination, with a multiplicative error model. The parameter estimates are presented in Table IV. There was no time-dependent PK behavior observed between exposure at day 1 and day 4, and data after single and repeated dosing could be well reproduced by the model (Fig. 3a and b).

**Table IV:** Model estimates and CV% of the model describing M8891 PK/PD in mouse

Parameter	Unit	Model estimate	Estimate CV (%)
ka	1/h	2.7	Fixed
CL/F	L/h/kg	0.415	13.8
V/F	L/kg	1.034	15.7
k_e0_	1/h	0.0566	7.3
K_in_	µg/mg/h	29.1	22
K_out_	1/h	1.45	25.7
I_max_	Unitless	0.91	1.3
IC_50_	ng/mL	340	17

PD data (Met-EF1α) from tumor tissue could be well described by an effect compartment combined with a turnover model with inhibition of the degradation of the biomarker as described in Eqs. (5) and (6). A good overlay with the observed PD levels was obtained both with the single-dose data and repeated-dose data from studies PK/PD-01 and PK/PD-02 (Fig. 3c and d). The corresponding parameters are listed in Table IV. Values for IC_50_ and I_max_ were estimated to be 340 ng/mL (or 0.88 μM total, 28 nM free) and 0.91, respectively.

As a confirmation step for the PK/PD model, PD modulation at the doses given in the efficacy study EFF-01 was simulated using the fitted PK/PD model. The predicted PD overlaid well with the observed PD data measured in tumor tissue extracted on day 42, after the last dose of continuous daily treatment (Fig. 3e). Thus, the model was validated and could be used to simulate the PD modulation in other efficacy studies in mice bearing the same tumor xenograft and would permit identification of the PD modulation level associated with anti-tumor efficacy.

**Fig. 4:**
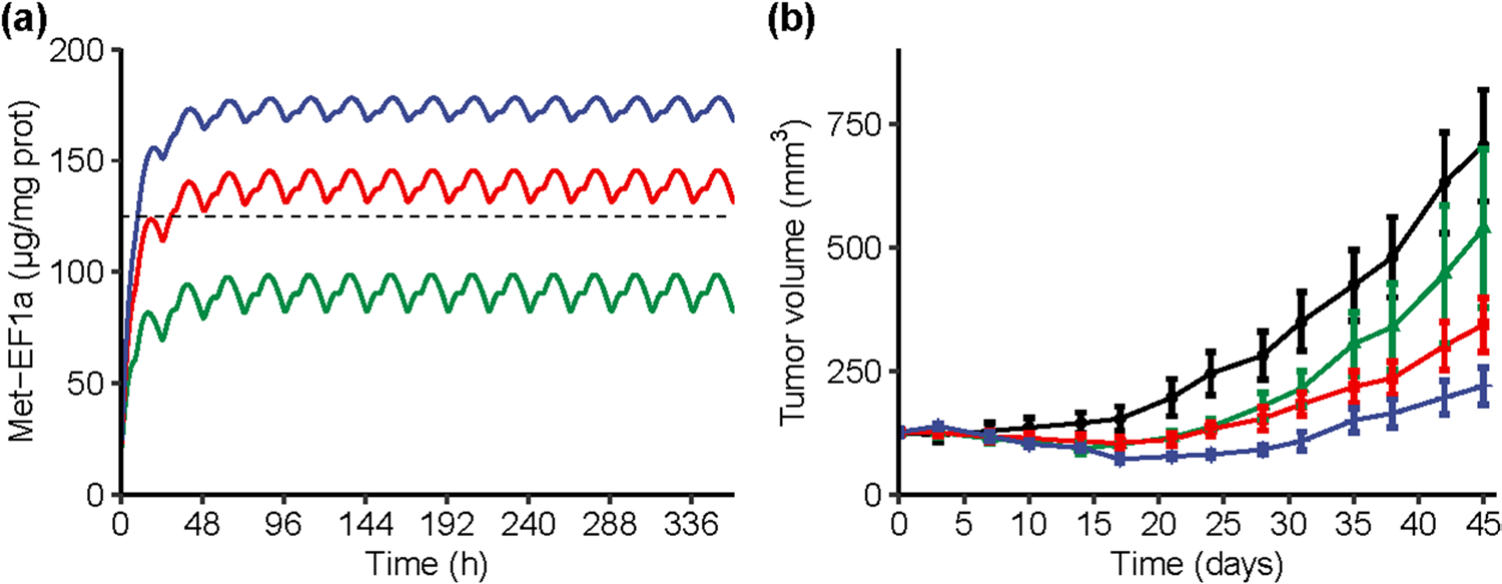
**(a)** Simulation of Met-EF1α in Caki-1 xenografts in mice treated orally with M8891 at 10 mg/kg BID (green), 25 mg/kg BID (red), or 50 mg/kg BID (blue). **(b)** Observed tumor volumes of Caki-1 xenografts in mice treated orally with M8891 at 10 mg/kg BID (green), 25 mg/kg BID (red), 50 mg/kg BID (blue), or vehicle (black), study EFF-02

In the separate efficacy study EFF-02 performed using the same xenograft cell line as used for the establishment of the PK/PD relationship, 25 mg/kg BID was identified as the minimal efficacious dose of M8891 in Caki-1 xenografts based on statistical comparisons across treatment groups (Fig. 4b). Simulation of the Met-EF1α modulation at the dose of 25 mg/kg BID (Fig. 4a) showed that at steady-state, the Met-EF1α ratio was maintained above a level of 125 µg/mg of total protein. Therefore, this level was selected as the target associated with anti-tumor efficacy in the mouse tumor xenograft model.

#### Human PK and PD Simulations

The simulations of the human oral C/t profile were based on predicting the ascending arm (first-pass effect and absorption process, i.e. F and k_a_) via PBPK modeling only, while for the prediction of the terminal arm all methods were equally weighted and the average values for CL and Vss were used and parameterized as 1-compartment model.

**Fig. 5:**
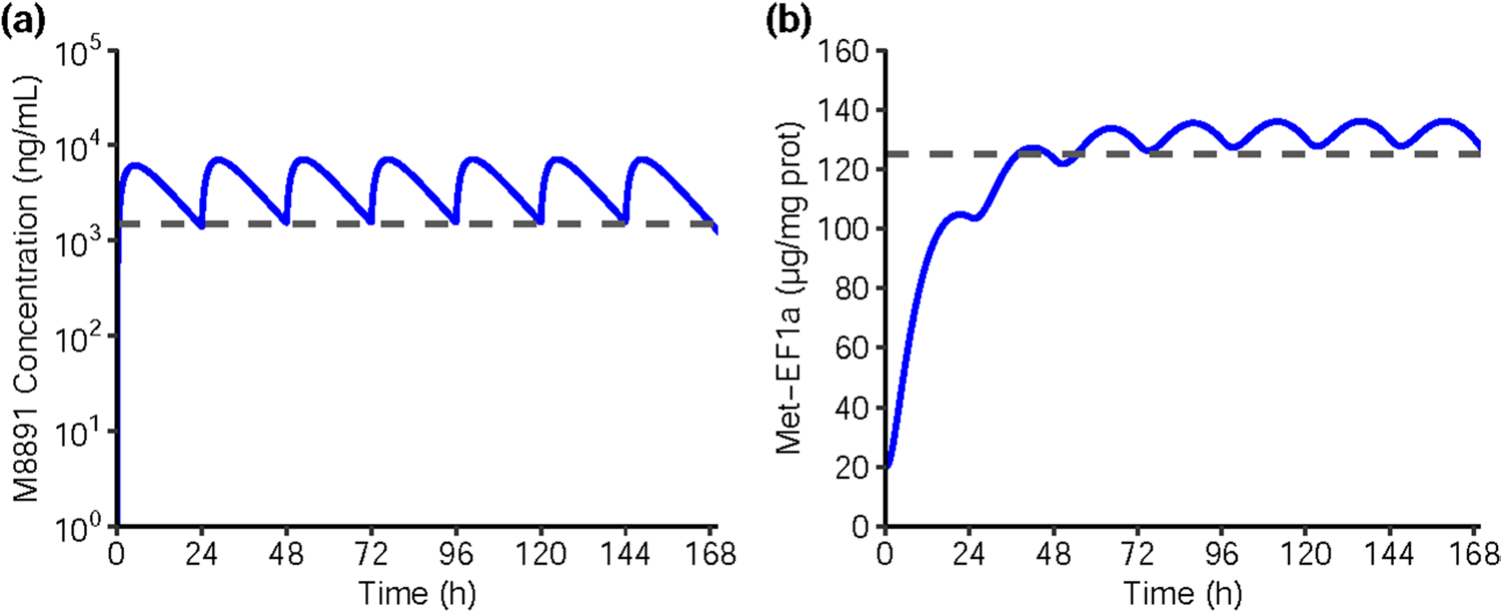
**(a)** Simulated M8891 plasma concentration in humans at repeated daily doses of 150 mg (blue line) using the predicted human PK parameters, overlaid with the C_trough_ predicted at this dose (grey dashed line). **(b)** Simulated Met-EF1α in tumor tissue in humans at repeated daily doses of 150 mg (blue line) using the preclinical PK/PD model coupled to the predicted human PK, overlaid with the target PD level associated with anti-tumor efficacy in the preclinical mouse xenograft model (grey dashed line)

The predicted human PK parameters were combined with the PK/PD model established from the mouse xenograft data in order to evaluate expected PK and PD modulation after M8891 administration in humans. Simulations showed that a dose of 150 mg QD would yield a relative Met-EF1α to total protein ratio above the target threshold of 125 µg/mg of total protein. In addition, a minimal M8891 concentration at steady-state of 1500 ng/mL (3.9 μM) was predicted (Fig. 5).

## Discussion

MetAP2 plays a key role in angiogenesis, and its inhibition is linked to antitumoral and antiangiogenic effects [2, 3]. M8891, which belongs to a novel class of selective and reversible MetAP2 inhibitors, has been shown to have strong anti-tumor activity *in vivo* [10].

To support the design of a Phase Ia dose escalation study of M8891 (NCT03138538), *in vitro* and *in vivo* preclinical data were leveraged to predict the PK characteristics of M8891 in humans. The human PK parameters were predicted using standard allometry, *in vitro* to *in vivo* extrapolation (IVIVE) methods, and PBPK modeling. Both for clearance and volume of distribution, the methods used led to estimated values in a narrow range, giving confidence in the reliability of the predictions. PBPK simulations, using a model first calibrated on preclinical PK data, were then utilized to predict the oral absorption profile of M8891 in humans over a range of doses.

In a preliminary approach using data from a panel of patient-derived xenografts in mice [10], an efficacious concentration range of 120 to 700 ng/mL (0.3 to 1.8 µM) M8891 in mouse was estimated, showing a clear dose–response relationship (data not shown). A renal cell carcinoma (RCC) model was used because RCC has a typically low p53 mutation rate and a strong angiogenic component driving tumor growth. Thus, RCC presents a promising opportunity to demonstrate the clinical activity of M8891 and provide proof of concept, potentially in combination with TKIs, which are the standard of care in this indication. The efficacious concentration range in mouse models was translated to 210 to 1240 ng/mL (0.5 to 3.2 µM) M8891 in humans by correcting for inter-species differences in protein binding between mice and humans. Based on predicted human PK parameters, simulations showed that an oral dose range of 20 to 100 mg daily would maintain the above-mentioned concentration range in humans.

In an effort to refine this prediction, a PK/PD model was established to describe the relationship between M8891 plasma exposure and modulation of the PD biomarker Met-EF1α in tumor tissue, based on data from xenografted mice. The identified IC_50_, 340 ng/mL, or 28 nM free, is comparable to the IC_50_ measured *in vitro* in Caki-1 cells [10]. However, the selected PK/PD model includes an effect compartment, which complexifies the interpretation of the estimated parameters, because it may not correspond to a real biological compartment. In this sense, the estimated IC_50_ does not necessarily correspond to the actual concentration at the target site. Nonetheless, simulations of the PD response at efficacious doses allowed identification of a target PD level associated with anti-tumor activity.

As the preclinical PD biomarker data were generated from human tumor cell xenografts, it was hypothesized that a similar PK/PD relationship could be observed in humans, and that the same level of biomarker modulation would be associated with anti-tumor efficacy. Based on these hypotheses, the Met-EF1α PD response upon M8891 treatment in humans was simulated, and a daily dose of 150 mg was calculated to be required to achieve the target PD level. This dose corresponds to a M8891 minimal concentration at steady-state of 1500 ng/mL (3.9 µM).

Use of population approaches such as non-linear mixed effects models was investigated during the establishment of the PK/PD model, but no proper model convergence or acceptable parameter estimation could be achieved, likely due to the low amount of data relative to the observed inter-individual variability. Thus, the selected modeling strategy was fit-for-purpose, tailored to the amount of data available, and nonetheless allowed a reasonable human translation of M8891 exposure and expected PD response on Met-EF1α.

The human dose predictions presented here should be considered cautiously, because they are based on predicted PK parameters and on target levels at steady-state. Therefore, small errors in the predicted clearance can markedly impact the resulting predicted dose [25]. Additionally, the median protein binding in mouse and human plasma was considered for all calculations, based on the assumption that the M8891 concentration levels at efficacious doses are in the concentration range of 5 µM, at which the median plasma protein binding was determined *in vitro*. Thus, including a function in the modeling to describe the limited concentration-dependency in plasma protein binding of about twofold minimally impacted the calculations.

M8891 exposure in humans at doses ranging from 7 to 80 mg daily was investigated in a Phase Ia dose escalation study (NCT03138538). The predicted human PK parameters were used to define the escalation steps in this study, while information from nonclinical toxicology studies was used to define the starting dose, following the recommendations of the ICH-S9 Guidance Document. The terminal half-life after oral administration was ≈30 h [26, 27] and thus four-fold higher than predicted, which is likely due to an overprediction of clearance in humans. This deviation can be partially explained by the prediction of human i.v. parameters, while – in the absence of an i.v. arm – only oral PK parameters, which are impacted by the absorption process, could be estimated from the Phase Ia study. In addition, human CL of M8891 is likely in the very low range of only ≈0.5–2% of liver blood flow, and thus, any small deviation of the scaled parameters is expected to strongly impact the CL prediction. In that respect, it should be noted that *in vitro* data obtained in human hepatocytes alone would have overpredicted the human clearance by only twofold. Moreover, human CL and V_ss_ predicted via PBPK modeling alone yielded a predicted terminal half-life of ≈14 h, only ≈twofold lower than the actual, observed half-life. For low clearance compounds such as M8891, classical *in vitro* systems may fail to appropriately capture the metabolic stability, as the activity of the drug metabolizing enzymes declines within a few hours [28]. This creates challenges for an accurate prediction of human clearance from *in vitro* measurements, with a poor correlation between predicted and observed clearance [29].

In addition, PD target levels were determined based on preclinical data to allow an assessment of the relevance of the administered doses in terms of target inhibition during the Phase I clinical study and to determine the RP2D. However, the need to measure biomarker modulation in tumor tissue, an invasive procedure for patients with solid tumors, severely limits sampling frequency and consequently hinders the establishment of a PK/PD model in humans. In the clinical Phase Ia dose escalation study, tumor biopsies were scheduled at screening and after dosing on the first day of the second treatment cycle (C2D1) to assess the modulation of Met-EF1α at expected steady-state. While such a limited dataset did not allow confirmation of the accuracy of the PK/PD relationship, clear dose/exposure-dependent target engagement, as shown by the ratio between Met-EF1α level at C2D1 compared to the level at screening, was observed [26]. Moreover, M8891 doses of 35 mg and higher led to PD modulation above the target level of 125 Met-EF1α µg per mg protein [26, 27].

A more detailed evaluation of the first-in-human data, including the RP2D, is described in Carducci *et al.*, 2023 [27]. Briefly, the observations in humans led to the definition of a RP2D of 35 mg M8891, lower than the efficacious dose predicted based on preclinical data presented here. The main cause for this discrepancy was the under-prediction of the human half-life, leading to an over-prediction of the dose required to maintain exposure above the target efficacious level. Nonetheless, the target PK and PD levels determined from the translational work presented here were pivotal for the definition of the RP2D [27].

## Conclusion

We have described the modeling and simulation techniques that were used to leverage M8891 preclinical data and support the translation to the clinic. *In vitro* and *in vivo* data were used to predict the PK of M8891 in humans using IVIVC, allometry, and PBPK methods, thus supporting the design of the phase I clinical study. Additionally, modeling of PK/PD and efficacy data from human tumor cell xenografts in mice led to the determination of 125 µg/mg protein as the target level of PD biomarker Met-EF1α associated with M8891 efficacy, which was pivotal in the definition of the recommended phase II dose.

### Supplementary Information

Below is the link to the electronic supplementary material.

Supplementary file1 (DOCX 329 KB)

## Data Availability

Any requests for data by qualified scientific and medical researchers for legitimate research purposes will be subject to the healthcare business of Merck KGaA, Darmstadt, Germany’s (CrossRef Funder ID: 10.13039/100009945) Data Sharing Policy. All requests should be submitted in writing to the healthcare business of Merck KGaA, Darmstadt, Germany’s data sharing portal (https://www.emdgroup.com/en/research/our-approach-to-research-and-development/healthcare/clinical-trials/commitment-responsible-data-sharing.html). When the healthcare business of Merck KGaA, Darmstadt, Germany has a co-research, co-development, or co-marketing or co-promotion agreement, or when the product has been out-licensed, the responsibility for disclosure might be dependent on the agreement between parties. Under these circumstances, the healthcare business of Merck KGaA, Darmstadt, Germany will endeavor to gain agreement to share data in response to requests.
