# Supplementary material for: Preclinical Pharmacokinetics and Translational Pharmacokinetic/Pharmacodynamic Modeling of M8891, a Potent and Reversible Inhibitor of Methionine Aminopeptidase 2
Source: Pharm Res. 2023 Oct 5;40(12):3011–23. doi: 10.1007/s11095-023-03611-z (PMC10746753; doi:10.1007/s11095-023-03611-z)
Supplement: Supplementary file 1 — Supplementary file1 (DOCX 329 KB) [file 11095_2023_3611_MOESM1_ESM.docx]

# Supplementary Materials

# I. Bioanalytical Details

## Internal Standard

The Merck compound MSC2394809, a structural analog of M8891, was selected as internal standard for the bioanalysis.

## Calibration Standards and Quality Control Samples in Plasma

Calibration standards and quality control samples were prepared in pooled heparinised plasma. The total volume of the spiked analyte solution (in pure solvent) was limited to 5% of the plasma volume in order to maintain matrix integrity. Calibration samples were prepared by serial dilution whereas QC samples were prepared by spiking different concentrations of stock and working solutions into blank plasma.

Calibration and QC samples were prepared freshly at the beginning of the bioanalytical phase, split into aliquots and stored frozen at -20±5°C until analysis.

| Analyte | Designation | Concentration level |
| --- | --- | --- |
| M8891 | Calibrators | 1.00, 2.00, 8.00, 32.0, 125, 500, 1000 ng/mL |
|  | QC | 3.00 ng/mL (QCLow) |
|  |  | 50.0 ng/mL (QCMid) |
|  |  | 800 ng/mL (QCHigh) |
|  |  | 10000 ng/mL (QCdilute) |

## Plasma Sample Preparation

Plasma samples were prepared using the following protocol:

- Thaw samples (calibrators, QC and unknown samples) and centrifuge for 1 min at 10000 RPM.
- Transfer 20 μL plasma into the cavity of a deepwell plate 96
- Add 180 μL working solution of internal standard (10 ng/mL in acetonitrile)
- Close cavities, shake carefully (10 min, Monoshake Wariomag) and centrifuge (5 min at 4000 rpm, Heraeus Megafuge 1.0R
- Transfer 100 μL organic layer into cavity of a 1.0 mL deepwell plate 96 and stream with nitrogen to dryness.
- Add 100 μL acetonitrile/water (1+9, v+v)
- Close deep well plate with deep well mat 96 and shake well (Eppendorf Thermomixer)
- Put 96 deep well plate in autosampler and inject 8 μL of each sample

## Analytical Details

The UPLC-MS/MS method was carried out using the following equipment and conditions:

| Pump: | Waters Acquity binary pump including degasser | | | |
| --- | --- | --- | --- | --- |
| Autosampler: | Waters Acquity thermostatted sample organizer and sample manager | | | |
| Sample temperature | 10°C | | | |
| Column oven: | Waters Acquity column organizer | | | |
| Column: | Waters BEH C18, 2.1 x 50 mm, 1.7 µm | | | |
| Column oven temperature: | 40°C | | | |
| Mobile Phase A: | Acetonitrile | | | |
| Mobile Phase B: | 0.1% Formic Acid | | | |
| Gradient composition: |  | | | |
|  | Time (min) | Mobile Phase A (%) | Mobile Phase B (%) | Flow  (mL/min) |
|  | initial | 5 | 95 | 0.60 |
|  | 0.10 | 5 | 95 | 0.60 |
|  | 0.70 | 100 | 0 | 0.60 |
|  | 1.50 | 100 | 0 | 0.60 |
|  | 1.80 | 5 | 95 | 0.60 |
| Injection volume: | 8 µL (may be varied depending on instrument performance) | | | |
| Injection mode: | Partial loop with needle overfill | | | |
| Injection loop: | 20 µL | | | |
| Needle placement: | 0.0 mm from bottom | | | |
| Weak wash solvent: | Acetonitrile / water (1+9, v+v) | | | |
| Weak wash volume: | 500 µL | | | |
| Strong wash solvent: | Methanol | | | |
| Strong wash volume: | 500 µL | | | |
| Detector: | Applied Biosystems Triple Quadrupole MS-Detector API 4000 | | | |
| Software: | Applied Biosystems Analyst, Version 1.4.2 | | | |
| Ion source | Turbo ion spray | | | |
| Scan Type | MRM (MS/MS) | | | |
| Polarity: | Positive | | | |
| Pause time | 5 ms | | | |
| Q1 Resolution | Unit | | | |
| Q3 Resolution | Unit | | | |
| Retention times | | | | |
| MSC2492280A | ~0.76 min | | | |
| MSC2394809 | ~0.79 min | | | |
| Mass ranges (amu)^a^ | | | | |
| MSC2492280A | 386.1 → 214.9@ 20 ms | | | |
| MSC2394809 | 402.0 → 214.9 @ 20 ms | | | |
| Calculation of results | | | | |
| Concentration data | Represents free compound | | | |
| Calibration curve | Linear regression | | | |
| Weighting | 1/x^2^ | | | |
| Rounding | 3 significant figures | | | |
| Accuracy (%) | $= \frac{measured concentration}{nominal concentration} \times100$ | | | |
| Precision (%) | $= \frac{standard deviation of concentrations determined}{mean concentration determined} \times100$ | | | |

Samples were analyzed in batches. A single batch comprised samples from several animals, together with a series of calibration standards and QC samples. Blank and zero standard samples were included to assess the selectivity of the analytical method. The samples from individual animals were analyzed in profile order. One set of calibration samples was measured at the beginning and one at the end of the run. QC samples were analyzed following the first set of calibrators, in the middle of the run and prior to the second set of calibrators.

## Acceptance Criteria

Plasma samples were analyzed with a qualified bioanalytical assay. The acceptance criteria are described hereafter:

| Criterion | Acceptance limits |
| --- | --- |
| Calibration standards | - ±15% (±20% at LLOQ) from nominal. - Outliers excluded from regression - ≥64% of standards must pass - ≥5 concentration levels must pass - r≥0.990 |
| QCs | - ±15% from nominal - 5/9 must pass, at least one per level |
| Selectivity | - Interference for analyte ≤20% of LLOQ signal. In case LLOQ bias ≥20% calibration curve to be revised and affected samples to be reanalyzed - Interference for IS ≤5% of average batch response |

# II. Non-Compartmental Analysis

The PK characteristics of M8891 in the preclinical species were estimated from the i.v. concentration-time profiles.

Using the linear up/log down trapezoidal method, the area under the curve (AUC) and the area under the first moment curve (AUMC) were estimated. The clearance (CL) that then estimated as: $CL= \frac{Dose}{AUC}$

The mean residence time (MRT) was calculated as : $MRT= \frac{AUMC}{AUC}$

The volume of distribution at steady state (Vss) was computed as : $V_{ss}=MRT \times CL$

The elimination half-life (t1/2) was calculated from the slope of the log-linear concentration-time curve as : $t_{1/2}= \frac{ln(2)}{slope}$

For each species, oral bioavailability was calculated from the ratio of the dose-normalized AUC of the p.o. PK divided by the dose-normalized AUC of the i.v. PK

# III. Establishment of a PKPB Model of M8891 Using Preclinical Data and Simulation of Human PK

## Methods

The PBPK modeling of M8891 in preclinical species and in humans was performed using GastroplusTM version 9.5.

Unless experimentally determined and/or user-defined values were available, either in silico predicted (ADMET predictor) or default parameter settings of GastroPlus^TM^ (e.g. gut physiology, transit times, blood/organ flows etc.) were used for all simulations. The compound-specific parameters relative to physico-chemical properties, formulation and in vitro permeability are presented in Tables S1 and S2. Further model settings and parameters are presented in the System Parametrization section.

PBPK modeling, based on in silico and in vitro input parameters, was run as initial step followed by compartmental PK modeling using in vivo PK parameters such as CL and V_ss_ derived from curve fitting of the observed concentration vs. time (C/t) profiles with compartmental models obtained from i.v. dosing. The drug’s absorption was predicted based on the ACAT (Advanced Compartmental and Transit) model of GastroPlus^TM^.

The absorption model was validated using experimental C/t profiles obtained in rats and dogs after oral dosing of the drug. All simulations of human absorption and C/t profiles were performed with the intended dosage form ‘Powder in Capsules’ (PiC) and assuming a body weight of 70 kg for both approaches (PBPK and compartmental PK modeling).

Simulations of human absorption and disposition of M8891 were conducted for both fasted and fed state using the model that best described the observed C/t profile(s) in the rat and dog as outlined in the results section. The plasma half-life was derived from the simulated human PK (C/t) profile and the oral bioavailability at anticipated efficacious dose was determined from simulations as shown in the results section.

Table S1 Physico-Chemical and Formulation Properties of M8891

| MW g/mol | logD @pH 7.4 | logP | pKa | Solubility (mg/mL) | | | | Particle size µm | Dosage form Humans |
| --- | --- | --- | --- | --- | --- | --- | --- | --- | --- |
|  |  |  |  | Ref. sol. ^1)^ | SGF | FaSSIF | FeSSIF |  |  |
| 385.37 | 2.25 | 2.04 | - | 0.0305 | 0.0355 | 0.0500 | 0.0545 | 6.94±5.20 | PiC |

1): Measured in water (pH 7.3)

Table S2 Permeability of M8891 and Reference Drugs in Presence of Cyclosporine A

|  | Permeability | Permeability (×10^-6^ cm/s) | | Recovery (%) | |  |
| --- | --- | --- | --- | --- | --- | --- |
| Compound | (classification) | P_app_ (A→B) | P_app_ (B→A) | A→B | B→A | Efflux ratio |
| M8891 | High | 39.9 | 40.7 | 108 | 88.0 | 1.02 |
| Atenolol | Low | 0.08 | 0.49 | 79.1 | 76.9 | 6.37 |
| Pindolol | Medium/high | 28.1 | 37.4 | 104 | 95.5 | 1.33 |
| Propranolol | High | 38.7 | 39.2 | 92.4 | 85.6 | 1.01 |

## Results

### PBPK Modeling of the Preclinical Data

Aiming to establish a predictive model, the simulation of the i.v. C/t profiles in the preclinical species rat and dog was carried out first using the PBPK approach (fasted state) with unbound CL_int_ values from studies in liver microsomes and in hepatocytes, respectively. The predicted (*in vivo*) CL and V_ss_ in these species are shown in Table S3, in comparison to the observed results determined by non-compartmental analysis (NCA).

Table S3 Predicted vs. Observed Pharmacokinetic Parameters (Clearance and Volume of Distribution) of M8891 in Dogs and Rats

| Parameter | Dog | | Rat | |
| --- | --- | --- | --- | --- |
|  | Predicted  (PBPK modeling) | Observed  (NCA) | Predicted  (PBPK modeling) | Observed  (NCA) |
| CL (L/h/kg) | 0.027 ^1)^ / 0.016 ^2)^ | 0.0341 | 0.589 ^1)^ / 0.296 ^2)^ | 0.35 |
| Vss (L/kg) | 0.239 | 0.227 | 0.352 | 1.30 |

1): CLint in liver microsomes; 2) CLint in hepatocytes.

These data indicate that the in vivo CL could be reasonably described in both species using Clint values from liver microsomes as well as from hepatocytes. However, in rat the V_ss_ was predicted almost 4-fold lower via PBPK modeling as compared to the observed value estimated from experimental in vivo data via NCA. Accordingly, the simulation output by PBPK modeling shows some deviations in rats at later time points (Figure S1).


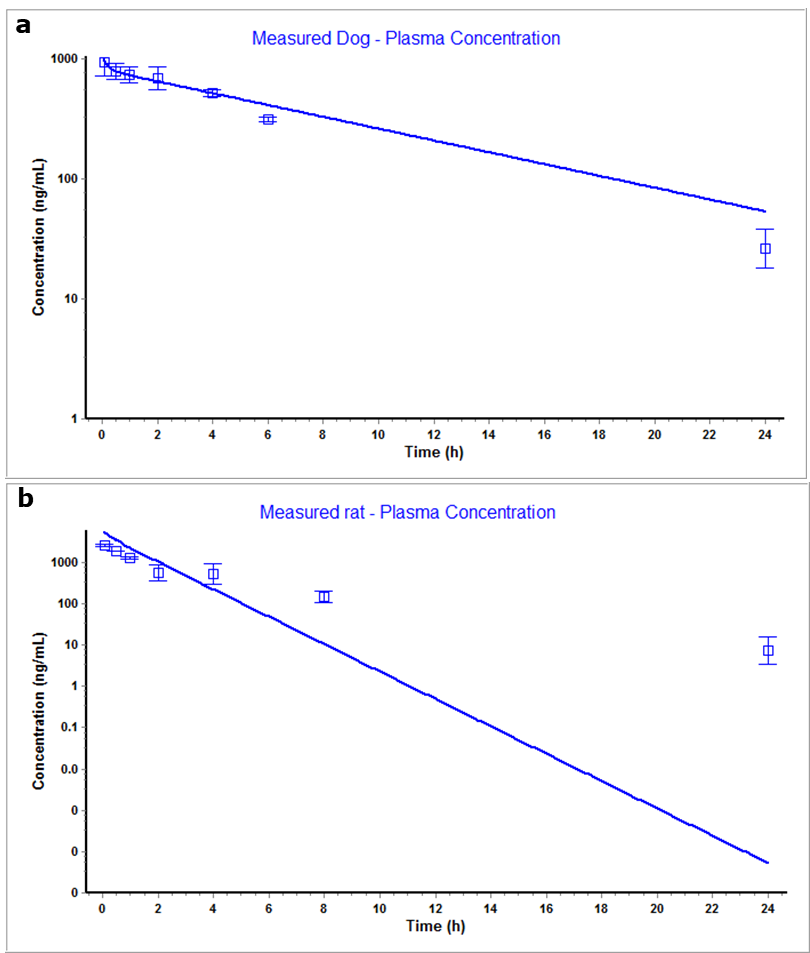


Figure S1. Simulated and Observed Intravenous Plasma Concentration vs. Time Profiles of M8891 in Dogs (a) and Rats (b) via PBPK Modeling

For further simulations via compartmental PK modeling in GastroPlus^TM^ in vivo PK data as input parameters were used, i.e. the mean i.v. concentration vs. time profiles of M8891 in dogs (Table S13) and rats (Table S15) were fitted based on a compartmental modeling approach (PKPlus^TM^ module) to generate PK parameters. As shown in Figure S2 and in Table S4, according to the Akaike Information Criterion (AIC: lowest number indicates the best-fit model), in dogs a 1-compartment model was found to describe the drug’s disposition with reasonable accuracy.


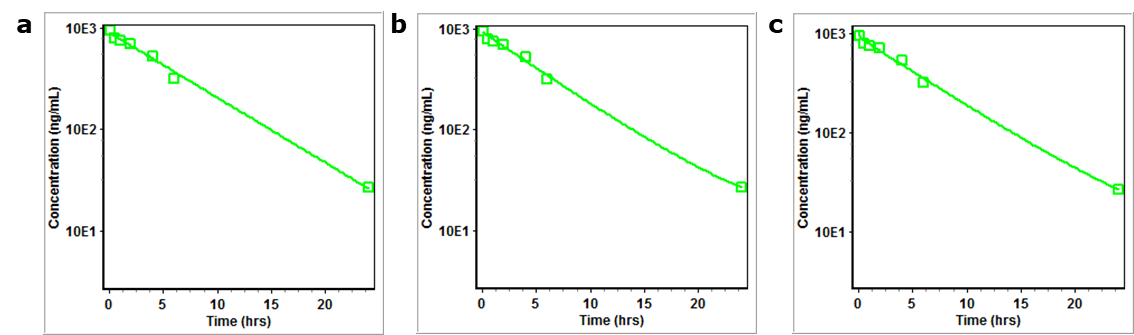


Figure S2. Intravenous Concentration vs. Time Profile of M8891 in Dogs Fitted with Compartmental Modeling. Points: observed data; lines: simulated concentration. a. One-compartment model; b. two-compartments model; c. three compartment model

In rats, the i.v. PK profile could be best described by a 2-compartment model; however, a 1-compartment model could also describe the experimental with reasonable precision (Table S4 and Figure S3).


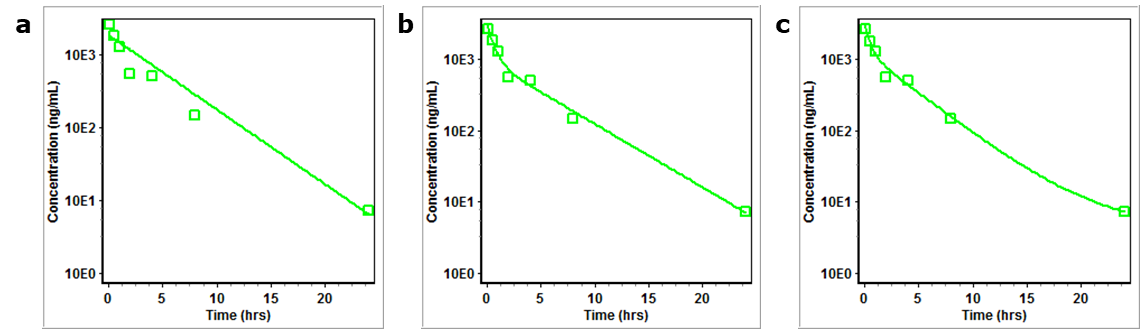


Figure S3. Intravenous Concentration vs. Time Profile of M8891 in Rats Fitted with Compartmental Modeling. Points: observed data; lines: simulated concentration. a. One-compartment model; b. two-compartments model; c. three compartment model

Table S4 Pharmacokinetic Parameters of M8891 in Dogs and in Rats Derived from Compartmental Modeling

| Parameter | 1-compartment | 2-compartment | 3-compartment |
| --- | --- | --- | --- |
| Dog |  |  |  |
| CL (L/h/kg) | 0.033 | 0.029 | 0.033 |
| Vc (L/kg) | 0.224 | 0.216 | 0.194 |
| AIC | -33.2 | -30.7 | -27.1 |
| Rat |  |  |  |
| CL (L/h/kg) | 0.256 | 0.344 | 0.346 |
| Vc (L/kg) | 1.084 | 0.670 | 0.668 |
| AIC | -10.9 | -19.2 | -16.8 |

Experimental in vivo data from the other two preclinical species mouse and monkey also suggested either a 1- or 2-compartment model (data not shown). For simplicity and even though in rats was not best described by a 1-compartment model, this model was further applied to simulate the oral C/t courses of M8891 at therapeutic dose level in order to evaluate whether or not the oral absorption process and the first-pass effect is correctly estimated. The predicted fraction absorbed (F_a_) and oral bioavailability (F) at the tested dose level in rats and dogs (Table S9 and Table S11 summarize the solubility and PSD data used as input) are compared to observed values (Table S5). Figure S4 shows the corresponding simulation results, compared to the observed oral profiles (Table S14 and Table S16 present the oral data in dog and rat, respectively). The simulation output indicated that predictions based on the compartmental approach described the observed oral concentration vs. time profile (C/t) with reasonable accuracy. Further simulations using solubility and PSD data of the batch representative for clinical development as input (Table S10 and Table S12) confirmed that the overall oral concentration time profile in rats could be reasonably well predicted with the established PK/ACAT model (data not shown). This model was therefore used for further simulations in humans.

Table S5 Predicted vs. Observed Fraction Absorbed and Oral Bioavailability of M8891 in Dogs and Rats

|  | Dog | | Rat | |
| --- | --- | --- | --- | --- |
|  | Predicted (Compartmental modeling) | Observed (NCA) | Predicted (Compartmental modeling) | Observed (NCA) |
| Parameter | Dose: 0.5 mg/kg | | Dose: 5 mg/kg | |
| F_a_ (%) | 100 | NA | 95 | NA |
| F (%) | 98 | 80 | 81 | 72 |


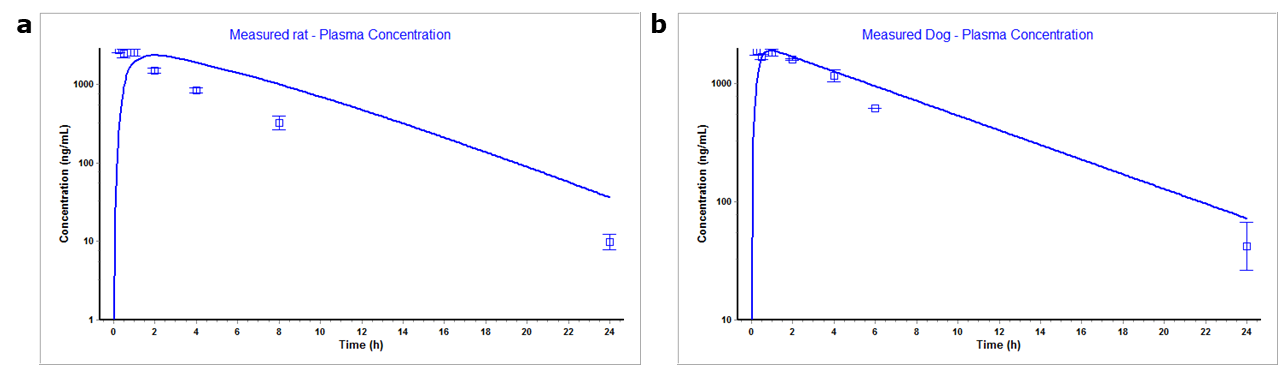


Figure S4. Simulated (using a 1-Compartment PK/ACAT Model) and Observed Oral Plasma Concentration vs. time Profiles in Rats (a) at 5 mg/kg, IR Suspension and in Dogs (b) at 0.5 mg/kg, IR Solution.

### Simulation of Oral Absorption and Concentration vs. Time Profiles in Humans

The simulations for human absorption and disposition of M8891 at a dose range from 50 to 400 mg in a PiC formulation were run with the compartmental approach previously established in rats and dogs. The predicted fractions absorbed under fasted and fed state conditions at 50 mg were similar (80 and 84%, respectively). The simulation outputs (t_max_, C_max_, C_min_, AUC_0-24h_, AUC_0-∞_, F_a_ and F) for one single dose of 50 mg in fasted and fed state are shown in Table S6. The corresponding C/t profiles are illustrated in Figure S5. Simulation results following repeated once (QD) daily oral administration in humans are summarized in Table S7. Thus, for the predicted efficacious dose of 150 mg QD (see main manuscript) oral bioavailability was predicted to be at least 60% and a rate of absorption (k_a_) was estimated to be 0.35 h^-1^, respectively. Additionally, the predicted human clearance and volume of distribution were 0.014 L/h/kg and 0.240 L/kg, respectively.


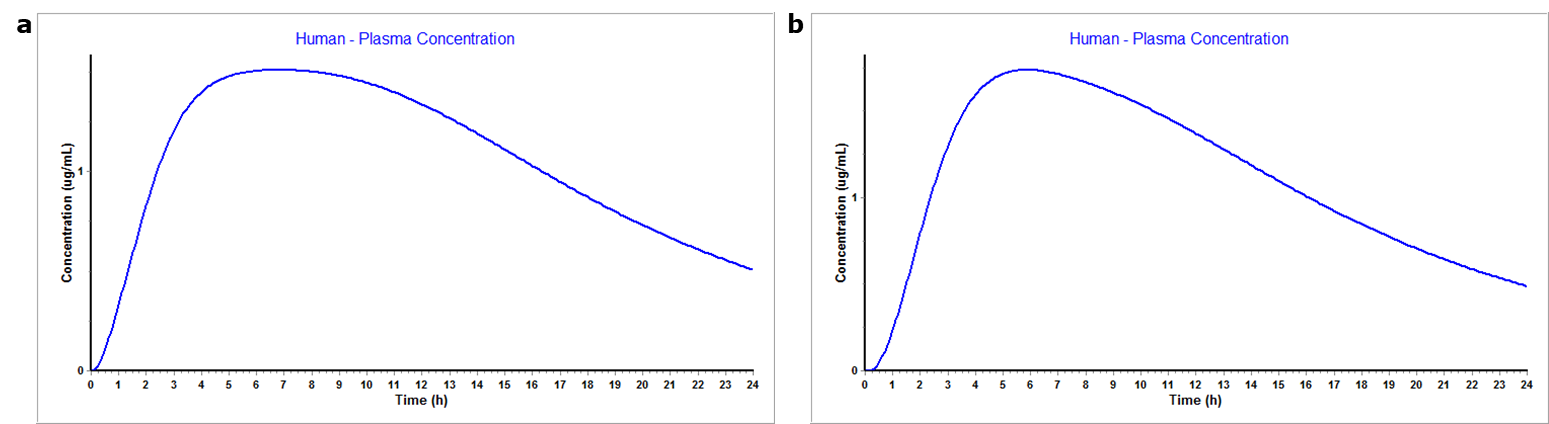


Figure S5. Simulated Oral Plasma Concentration vs. Time Profiles of M8891 in Humans Based on a 1-Compartmental/ACAT Model. (a) Fasted State Simulation; (b) Fed State Simulation.

Table S6 Predicted Human PK Parameters of M8891 After Single Oral Administration of 50 mg in Fed and Fasted State

| Parameter | | Fasted state | Fed state |
| --- | --- | --- | --- |
| t_max_ | (h) | 3.5 | 4.2 |
| C_max_ | (ng/mL) | 1920 | 1930 |
| C_min_ | (ng/mL) | 433 | 439 |
| AUC_0-24 h_ | (ng/mL × h) | 27100 | 27100 |
| AUC_0-∞_ | (ng/mL × h) | 32000 | 32000 |
| F_a_ | (%) | 94 | 95 |
| F | (%) | 89 | 89 |

Table S7 Predicted Human PK Parameters of M8891 Following 14 Days Repeated Oral Administrations

| Parameter | | 50 mg | 100 mg | 150 mg | 200 mg | 300 mg | 400 mg |
| --- | --- | --- | --- | --- | --- | --- | --- |
| t_max_ | (h) | 3.9 | 4.1 | 4.1 | 4.4 | 4.7 | 5.0 |
| C_max_ | (µg/mL) | 2.0 | 4.42 | 6.51 | 8.47 | 11.8 | 14.6 |
| C_min_ | (µg/mL) | 0.485 | 1.00 | 1.54 | 2.11 | 3.33 | 4.58 |
| AUC_0-24 h_ | (µg/mL x h) | 434 | 868 | 1310 | 1730 | 2510 | 3180 |
| F (%) | (%) | 86.5 | 78.9 | 70.8 | 62.8 | 49.4 | 40.5 |

C_max_ , C_min_ and AUC: maximum and minimum concentration of dosing interval at steady state, respectively.

## System Parameterization

Table S8 System and Default Parameter Settings

| Input tab | Parameter | Value or setting* |
| --- | --- | --- |
| Compound | Precipitation time | 900 s |
|  | Mean particle density | 1.2 g/mL |
|  | Mean particle radius | < 10 µm |
|  | Diffusion coefficient | Predicted (ADMET predictor) |
|  | Dissolution model | Johnson |
|  | Effect of temperature on solubility | 37°C |
|  | Nanoparticle effect | On (adjust solubility) |
|  | Bile salt effect | On (adjust solubility) |
|  | Diffusion layer thickness | 30 µm |
|  | Enterohepatic circulation | Off |
|  |  |  |
| Gut physiology | Excrete all un-absorbed drug at the end of transit time | Off |
|  | Zero-order gastric emptying | Off |
|  | Dog – physiological – fasted | Default |
|  | Dog – physiological – fed | Default |
|  | Liver blood flow (Qh) - Dog | Default |
|  | Human – physiological – fasted | Default |
|  | Human – physiological – fed | Default |
|  | Liver blood flow (Qh) - human | Default |
|  | ASF model | Opt –logD Model SA/V 6.1 |
|  |  |  |
| Pharmacokinetics | Body weight (compartmental PK model) | 70 kg |
|  | Population data (PBPK modeling) | HumanAmerican30YO_70 kg |
|  | Liver first pass extraction (compartmental PK model) | Calculated based on Cl input |
| * default values used unless stated otherwise | | |

Table S9 Particle Size Distribution of M8891 (Preclinical Batch)

| Mean radius (µm) | Standard deviation (µm) | Distribution (%) | Distribution type (log, log-normal) | Unit |
| --- | --- | --- | --- | --- |
| 10 | - | 62 | - | % Fraction |
| 29 | - | 19 | - | % Fraction |
| 49 | - | 7 | - | % Fraction |
| 68 | - | 2 | - | % Fraction |
| 126 | - | 3 | - | % Fraction |
| 146 | - | 7 | - | % Fraction |
| < 10 | NA | 100 | - |  |

Table S10 Particle Size Distribution of M8891 (Clinical Batch)

| Mean radius (µm) | Standard deviation (µm) | Distribution (%) | Distribution type (log, log-normal) | Unit |
| --- | --- | --- | --- | --- |
| 11 | - | 40 | - | % Fraction |
| 33 | - | 43 | - | % Fraction |
| 55 | - | 8.4 | - | % Fraction |
| 77 | - | 0.7 | - | % Fraction |
| 99 | - | 1.4 | - | % Fraction |
| 165 | - | 6.7 | - | % Fraction |
| 19.4 | NA | 100.2 | - |  |

Table S11 Solubility Profile (pH-Dependent) of M8891, Preclinical Batch

| pH | Solubility (mg/mL) | Dose dissolved in 250 mL (mg) |
| --- | --- | --- |
| 1.2 | 0.0269 | 6.725 |
| 5.0 | 0.0645 | 16.125 |
| 6.5 | 0.0535 | 13.375 |

Table S12 Solubility Profile (pH-Dependent) of M8891, Clinical Batch

| pH | Solubility (mg/mL) | Dose dissolved in 250 mL (mg) |
| --- | --- | --- |
| 1.2 | 0.0355 | 8.875 |
| 5.0 | 0.0545 | 13.625 |
| 6.5 | 0.05 | 12.5 |

Table S13 Plasma Concentration vs. Time Profiles of M8891 After Intravenous Administration to Dog

| Experimental conditions | | Time (h) | Concentration (ng/mL) | CV% |
| --- | --- | --- | --- | --- |
| Species | Dog | 0.1 | 940 | 25.7 |
| Strain | Beagle | 0.5 | 789 | 15.1 |
| Gender | Female | 1 | 747 | 15.9 |
| Dose (i.v.) | 0.2 mg/kg | 2 | 697 | 22.2 |
| Body weight | 8.36 kg | 4 | 525 | 6.73 |
| No. of animals | N=2 | 6 | 316 | 3.81 |
| Formulation | IR: solution | 24 | 26.4 | 36.4 |

Table S14 Plasma Concentration vs. Time Profiles of M8891 After Oral Administration to Dogs

| Experimental conditions | | Time (h) | Concentration (ng/mL) | CV% |
| --- | --- | --- | --- | --- |
| Species | Dog | 0.25 | 1870 | 8.72 |
| Strain | Beagle | 0.5 | 1680 | 4.70 |
| Gender | Female | 1 | 1800 | 5.91 |
| Dose (p.o.) | 0.5 mg/kg | 2 | 1580 | 1.35 |
| Body weight | 7.93 kg | 4 | 1160 | 11.6 |
| No. of animals | N=2 | 6 | 613 | 0.577 |
| Formulation | IR: solution | 24 | 42.1 | 47.0 |

Table S15 Plasma Concentration vs. Time Profiles of M8891 After Intravenous Administration to Rat

| Experimental conditions | | Time (h) | Concentration (ng/mL) | CV% |
| --- | --- | --- | --- | --- |
| Species | Rat | 0.1 | 2620 | 4.47 |
| Strain | Wistar | 0.5 | 1810 | 1.15 |
| Gender | Male | 1 | 1270 | 7.76 |
| Dose (i.v.) | 2 mg/kg | 2 | 556 | 46.2 |
| Body weight | 0.270 kg | 4 | 510 | 57.2 |
| No. of animals | N=3 | 8 | 146 | 32.7 |
| Formulation | IR: solution | 24 | 7.29 | 74.2 |

Table S16 Plasma Concentration vs. Time Profiles of M8891 After Oral Administration to Rat

| Experimental conditions | | Time (h) | Concentration (ng/mL) | CV% |
| --- | --- | --- | --- | --- |
| Species | Rat | 0.25 | 2760 | 7.35 |
| Strain | Wistar | 0.5 | 2500 | 12.8 |
| Gender | Male | 1 | 2560 | 10.2 |
| Dose (p.o.) | 5 mg/kg | 2 | 1520 | 7.89 |
| Body weight | 0.273 kg | 4 | 843 | 7.25 |
| No. of animals | N=3 | 8 | 329 | 20.0 |
| Formulation | IR: suspension | 24 | 9.82 | 22.3 |
